# Injuries of Joints

**Published:** 1894-10-13

**Authors:** 


### Injuries of Joints.

The management of traumatic lesions of the articulations has given rise to much discussion amongst surgeons. Difficult of diagnosis, troublesome in treatment, the source of much discontent on the part of patients, these cases are often the most tiresome with which we have to deal. When authorities differ upon some of the most important points connected with this subject, it becomes exceedingly hard for the practitioner to decide what lines to follow. If he waver he may lose a golden opportunity; if undecided he may be discarded in favour of the bone-setter or the quack. This being so, we are sometimes compelled to fall back upon principles, the foundation of all good and conscientious work, rather than hard and fast rules, which, even in the hands of the most expert, are often destined to failure. Nowhere is the need of such a guide more felt than in the treatment of injuries to the articulations. The chief structures we have to consider in connection with a joint are the bones, the ligaments which bind them together, and the muscles which move them at the articulation. The importance of these structures in their relation to the management of these cases can hardly be over-estimated, for it is upon the physical and anatomical sources of joint strength and weakness that our method of treatment must largely depend. These last are not without their influence upon the nature of the injury. Thus the osseously-strong elbow is especially liable to fracture; the ligamentously-strong radio-ulnar articulation is more prone to sprain. As a muscularly-strong joint we have the shoulder, and here we find a tendency to dislocation. Amongst physical characters of joints we may mention the proximity of numerous joints constituting a source of strength to the wrist. The force resulting from a fall upon the hand is dispersed; while the wrist may thus escape, the same force may be concentrated upon the lower end of the shaft of the radius, causing this bone to give way. This, again, is a source of strength to the wrist, just as fracture of the fibula may save the ankle-joint. In a joint such as the knee the presence of cartilage greatly lessens the effect of a shock or blow. Great mobility may be a source of weakness, and much may depend upon the leverage a joint has to bear.

Atmospheric pressure is said to have some influence, and the position in which a joint is placed at the time will largely determine the character of an injury. We repeat, then, that it is upon the physical and anatomical sources of joint strength and weakness that our method of treatment must largely depend. In sprain of a ligamentously-strong joint, more harm may result from too prolonged rest than from too early movement. Indeed, early movement is most important here. When effusion is removed by accurate elastic pressure, massage and movement should be begun as soon as the patient can bear it. Far better thus to prevent adhesions than, with more prolonged rest, to require to break them down. It is far otherwise in a contusion, where rest is absolutely essential, on account of the danger of destruction of the joint by disease, deep seated, it may be, in cartilage or bone.

In dislocation of a ligamentously-strong joint, after reduction, we can hardly keep the part too long quiet; whereas in a joint that is osseously strong one can scarcely begin movement too soon.

These are some of the points which will guide us in the management of these important cases, and there can be little doubt that more might be made of them, both by teachers and taught, when engaged in the study of anatomy. Anatomy is as essential to the Art as it is to the Science of surgery, and a closer alliance—a greater continuity between the study of anatomy and that of surgery, would do much to create a greater unanimity of opinion on many questions of this kind, and must have an influence towards the acquirement of what Greig Smith has so aptly termed "brain-craft uttered through the fingers."

